# Diagnosing and Treating Inflammatory Myofibroblastic Tumor of the Bladder

**DOI:** 10.1155/2016/5724020

**Published:** 2016-05-18

**Authors:** Ridwan Alam, Michael H. Johnson, Trevor Caldwell, Christian P. Pavlovich, Trinity J. Bivalacqua, Jeffrey J. Tosoian

**Affiliations:** James Buchanan Brady Urological Institute, The Johns Hopkins University School of Medicine, Baltimore, MD 21287, USA

## Abstract

Inflammatory myofibroblastic tumor (IMT) is an uncommon condition that is rarely encountered in the urinary tract. In this report, we present a case of IMT of the bladder in a woman with multiple previous pelvic surgeries. We further review the relevant literature to highlight this rare but important clinical presentation.

## 1. Introduction

The vast majority of bladder neoplasms are malignant, with urothelial (transitional cell) carcinoma representing over 90% of all bladder cancers worldwide, particularly in the United States and Europe [1]. In other regions of the world, nonurothelial carcinomas are more common. A relatively high incidence of nonurothelial bladder cancer is seen in East Africa and the Middle East due to schistosomiasis [1]. Benign neoplasms, on the other hand, are much rarer and have typically been of little consequence due to the relative ease with which they are distinguished from malignancy via biopsy.

IMT presents a unique diagnostic challenge because of the characteristics it shares with malignant neoplasms. As a spindle cell lesion that has eluded the understanding of the medical community since the 1980s, IMT is a disease of unclear malignant potential. Consequently, many different names have been assigned to this condition, including inflammatory pseudotumor, nodular fasciitis, and pseudomalignant spindle cell proliferation [2, 3]. Here, we present the case of a woman who initially presented with gross hematuria and was later determined to have IMT of the bladder.

## 2. Case Presentation

**Figure 1:**
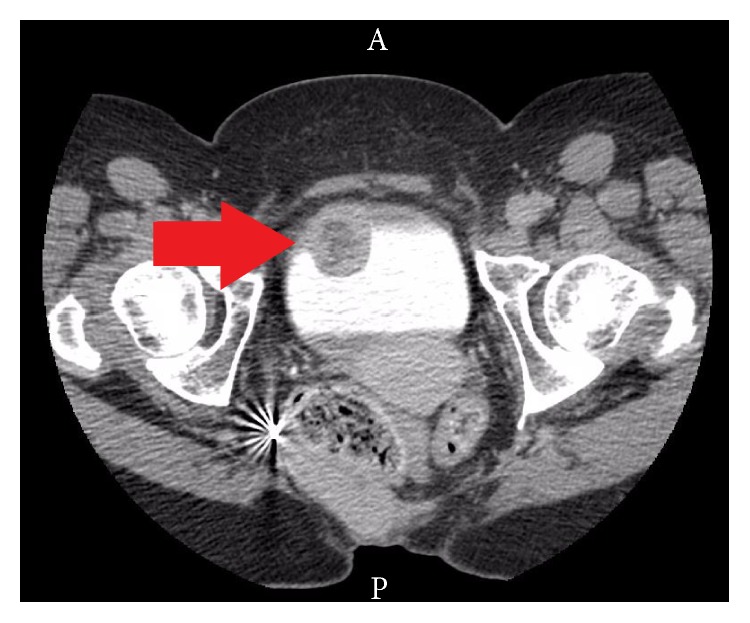
CT with contrast of the abdomen and pelvis demonstrating 3.2 cm anterior bladder mass (red arrow). The mass is a smooth, well-circumscribed lesion, which would be atypical for urothelial cell carcinoma.

A 39-year-old woman presented to her gynecologist complaining of vaginal bleeding four days after a regular menstrual cycle. Her relevant medical history included multiple congenital anomalies in the setting of cerebral palsy. Previous surgeries included sacrococcygeal teratoma resection, transverse vaginal septectomy, and neourethra in the vagina. An ultrasound of the pelvis was performed to evaluate the cause of her bleeding. Imaging displayed a mass arising from the right anterior bladder wall. Her hemoglobin at this time was 11.3 g/dL. A CT scan of the abdomen and pelvis confirmed the presence of a 3.2 cm × 2.0 cm × 2.7 cm mass with no lymphadenopathy or distant metastasis (Figure 1).

**Figure 2:**
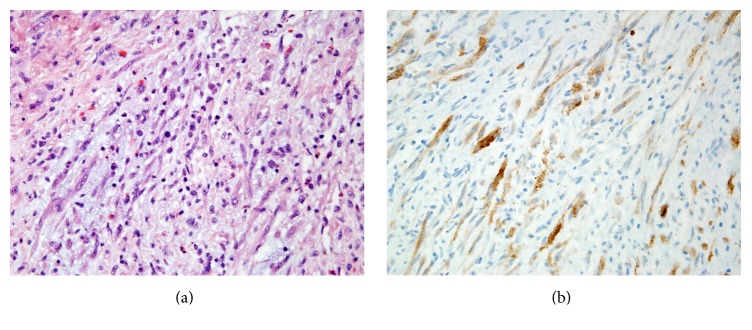
(a) Hematoxylin and eosin stain of the bladder mass showing features consistent with IMT. Spindle cells and nuclear atypia are seen in a background of mixed inflammation with variable myxoid stroma. (b) ALK immunohistochemistry of the bladder mass showing cytoplasmic positivity in spindle cells. The identification of ALK suggests a neoplastic process and distinguishes IMT from other spindle cell lesions, which do not express ALK. The ALK protein is detectable in about 50% of IMTs.

Ten days after her initial presentation, she presented to the Emergency Department with severe hematuria and associated dizziness and fatigue. She denied symptoms such as fever, chills, or weight loss. Her hemoglobin level at this time was 4.8 g/dL. Blood transfusion and continuous bladder irrigation (CBI) were initiated. Cystoscopy was performed and the bladder mass was biopsied. Pathologic examination of the biopsy was consistent with IMT (Figure 2(a)). After two days of CBI, her hemoglobin stabilized at 9.1 g/dL.

She returned to the operating room for complete transurethral resection of her bladder mass. Pathologic review confirmed an IMT involving the muscularis propria. Immunohistochemical staining was positive for anaplastic lymphoma kinase (ALK), consistent with the diagnosis of IMT (Figure 2(b)). Cystectomy was considered but ultimately deferred in favor of close follow-up. A surveillance cystoscopy, urine cytology, and CT scan of the abdomen and pelvis at three-month follow-up revealed no evidence of recurrent IMT.

## 3. Discussion

Inflammatory myofibroblastic tumors are uncommon lesions of unclear malignant potential. Although IMT as a whole more frequently affects individuals younger than 20 years of age, IMT of the urinary tract is most commonly observed in adults in their 40s and 50s [2–4]. Furthermore, males are more likely to present with this condition than females [2, 3].

IMTs have been reported in multiple anatomic locations, with the most frequent site being the lungs [4]. To this point, reports of IMT in the urinary tract have been limited. While the malignant potential of these lesions appears to vary based on site, IMTs of the bladder have been associated with a more indolent course in most cases. Although the etiology of urinary tract IMT is still unknown, current hypotheses based on previous reports include infection, trauma, or surgery [4]. Consistent with this theory, our patient presents with a history of multiple pelvic surgeries which may be the cause of her IMT.

When present in the urinary tract, IMT most frequently occurs in the bladder and presents with hematuria [2–4]. Radiographic imaging is of marginal benefit due to the nonspecific features this tumor shares with malignant neoplasms. As a result, biopsy of the mass becomes the cornerstone of this histologically defined disease. Under microscopic examination, IMT characteristically displays myofibroblastic spindle cells with inflammatory components [4]. Sarcomatoid urothelial carcinomas can exhibit features mimicking IMT or present concurrently with IMT, compounding the difficulty of making an accurate diagnosis. Unlike IMT, previous studies have shown that sarcomatoid carcinomas do not express ALK on immunohistochemistry, but further investigation is needed to confirm these findings [2, 3, 5].

Treatment often involves only local resection of the tumor. Even after incomplete resection, urinary IMTs tend to regress or remain stable [3]. Recurrence occurs in 10–25% of patients and generally follows an indolent course [2, 3]. Malignant IMT, defined as histologic transformation of spindle-shaped cells to atypical polygonal cells with atypical mitoses in the setting of classic IMT immunohistochemistry profiles (i.e., positive for ALK and negative for myoglobin and S-100), is very rare but necessitates further intervention [5]. However, no standardized protocol has been established for the treatment of malignant IMT. Instead, bladder cancers are classically treated based on the known natural history of urothelial carcinoma, which may not be applicable to malignant IMT. A recent case of malignant, nonmetastatic IMT of the bladder in a 14-year-old boy was treated with three cycles of systemic chemotherapy alternating with two cycles of internal iliac arterial infusion chemotherapy before radical resection of the bladder mass with 0.8 cm margins. Postoperatively, the patient received nine cycles of systemic combined chemotherapy, and no recurrence was detected 15 months after initial diagnosis [5]. Despite the rarity of recurrence, IMTs merit close follow-up due to their unknown malignant potential and the difficulty in excluding the diagnosis of sarcomatoid carcinoma.

## 4. Conclusion

This report describes a rare case of bladder IMT, which usually follows a relatively indolent course. However, it can be difficult to distinguish IMT from sarcomatoid carcinoma both clinically and histologically. As such, local surgical resection with close follow-up remains the mainstay of treatment for urinary tract IMT.

## Abbreviations

IMT: Inflammatory myofibroblastic tumor
CBI: Continuous bladder irrigation
ALK: Anaplastic lymphoma kinase.
